# Transcriptome profiling analysis of sex-based differentially expressed mRNAs and lncRNAs in the brains of mature zebrafish (*Danio rerio*)

**DOI:** 10.1186/s12864-019-6197-9

**Published:** 2019-11-08

**Authors:** Wenliang Yuan, Shouwen Jiang, Dan Sun, Zhichao Wu, Cai Wei, Chaoxu Dai, Linhua Jiang, Sihua Peng

**Affiliations:** 1 Key Laboratory of Exploration and Utilization of Aquatic Genetic Resources (Shanghai Ocean University), Ministry of Education, Shanghai, 201306 China; 2 National Pathogen Collection Center for Aquatic Animals, Ministry of Agriculture, Shanghai, 201306 China; 3 International Research Center for Marine Biosciences at Shanghai Ocean University, Ministry of Science and Technology, Shanghai, 201306 China; 4 School of Optical-Electric and Computer Engineering, University of Shanghai for Science and Technology, Shanghai, 200093 China; 5 College of Mathematics and Information Engineering, Jiaxing University, Jiaxing, 314001 China

**Keywords:** Zebrafish, Brain, The dimorphic patterns, mRNA and lncRNAs, High-throughput transcriptome sequencing

## Abstract

**Background:**

Similar to humans, the zebrafish brain plays a central role in regulating sexual reproduction, maturation and sexual behavior. However, systematic studies of the dimorphic patterns of gene expression in the brain of male and female zebrafish are lacking.

**Results:**

In this study, the mRNA and lncRNA expression profiles were obtained from the brain tissue samples of the three male and three female zebrafish by high-throughput transcriptome sequencing. We identified a total of 108 mRNAs and 50 lncRNAs with sex-based differential expression. We randomly selected four differentially expressed genes for RT-qPCR verification and the results certified that the expression pattern showed a similar trend between RNA-seq and RT-qPCR results. Protein-protein interaction network analysis, Gene Ontology (GO) analysis, and Kyoto Encyclopedia of Genes and Genomes (KEGG) analysis were performed to obtain the biological significance of differentially expressed mRNA in the brain dimorphism of zebrafish. Finally, a Pearson correlation analysis was performed to construct the co-expression network of the mRNAs and lncRNAs.

**Conclusions:**

We found that 12 new lncRNAs not only have significant gender specificity in the brain of zebrafish, and this finding may provide a clue to further study of the functional difference between male and female zebrafish brain.

## Background

The zebrafish (*Danio rerio*) is a very useful model animal for the comparative study of neuroscience [1], because its brain has behavioral and morphological sexual dimorphisms. Although the sex-related chromosomal regions of zebrafish are not fixed [2, 3] and their genome of different sexes can be very similar; we can observe its sexual dimorphism by differences in gene expression [4]. By using microarray technology, studies have found 24 gender differential gene expression in mature zebrafish brain [5]. The zebrafish exposed to the sex hormone showed a regional (forebrain, midbrain and hindbrain) and gender-related differences in gene expression [6]. The expression of 61 genes in the four zebrafish showed significant gender differences by RNA-seq sequencing data analysis [7]. Arslan-Ergul et al. also found that there are expression differences of various genes between genders and for different ages, which are associated with multiple pathways in zebrafish brain [8].

Long non-coding RNAs (long ncRNAs, lncRNAs), which may appear anywhere in the genome, are defined as transcripts longer than 200 nucleotides that are not translated into protein [9]. Non-functional lncRNAs are likely to be the result of transcriptional noise, whereas functional lncRNAs act in *cis* and/or in *trans* [10]. The over-expression or deficiency of lncRNAs is involved in numerous human diseases [11]. Guttman M et al. predicted that lncRNA plays an important role in cell pluripotency and cancer [12]. It was also found that some regulatory genes and lncRNAs may play a key role in development and hematopoiesis through processing functional coupling network using deep RNA-seq sequencing [13]. Durga, a novel non-coding RNA, arising from the first exon of Kalirin, is a key player in axonal development in zebrafish and maintains dendritic length and density by regulating kalrna expression [14]. Similarly, many lncRNAs with biological functions have been found in zebrafish [15–18]; however the function of the sex-based lncRNAs in the brain is still unknown.

In this study, we obtained the RNA expression data of the brains of the adult male and female zebrafish by using transcriptome sequencing (RNA-seq). Differential expression mRNAs and lncRNAs were screened using computational methods. Some of the differential expression mRNAs and lncRNAs have been verified by RT-qPCR. Additionally, we found the mRNAs significantly enriched in many pathways according to the GO and KEGG functional enrichment analyses. Finally, the lncRNA-mRNA interaction network was constructed using the Pearson correlation coefficients, based on the FPKM values of the lncRNAs and mRNAs. This study expanded the zebrafish brain sex-based lncRNA catalogue and constructed a regulatory network of the zebrafish brains at the transcriptional level, providing clues for more in-depth depiction of gender differences in zebrafish brain neurons.

## Results

### Sequencing data, raw data filtering, and mapping of RNA sequencing reads onto the zebrafish genome

High-throughput sequencing generated 113.71 G bp of raw data (Additional file 1), and after filtering, the clean data of 98.52 G bp were extracted. Then, these high-quality reads were mapped to the reference zebrafish genome by 89.2%. The uniquely mapped reads ranged from 78.3 to 83.4%.

### Differentially expressed genes

**Fig. 1:**
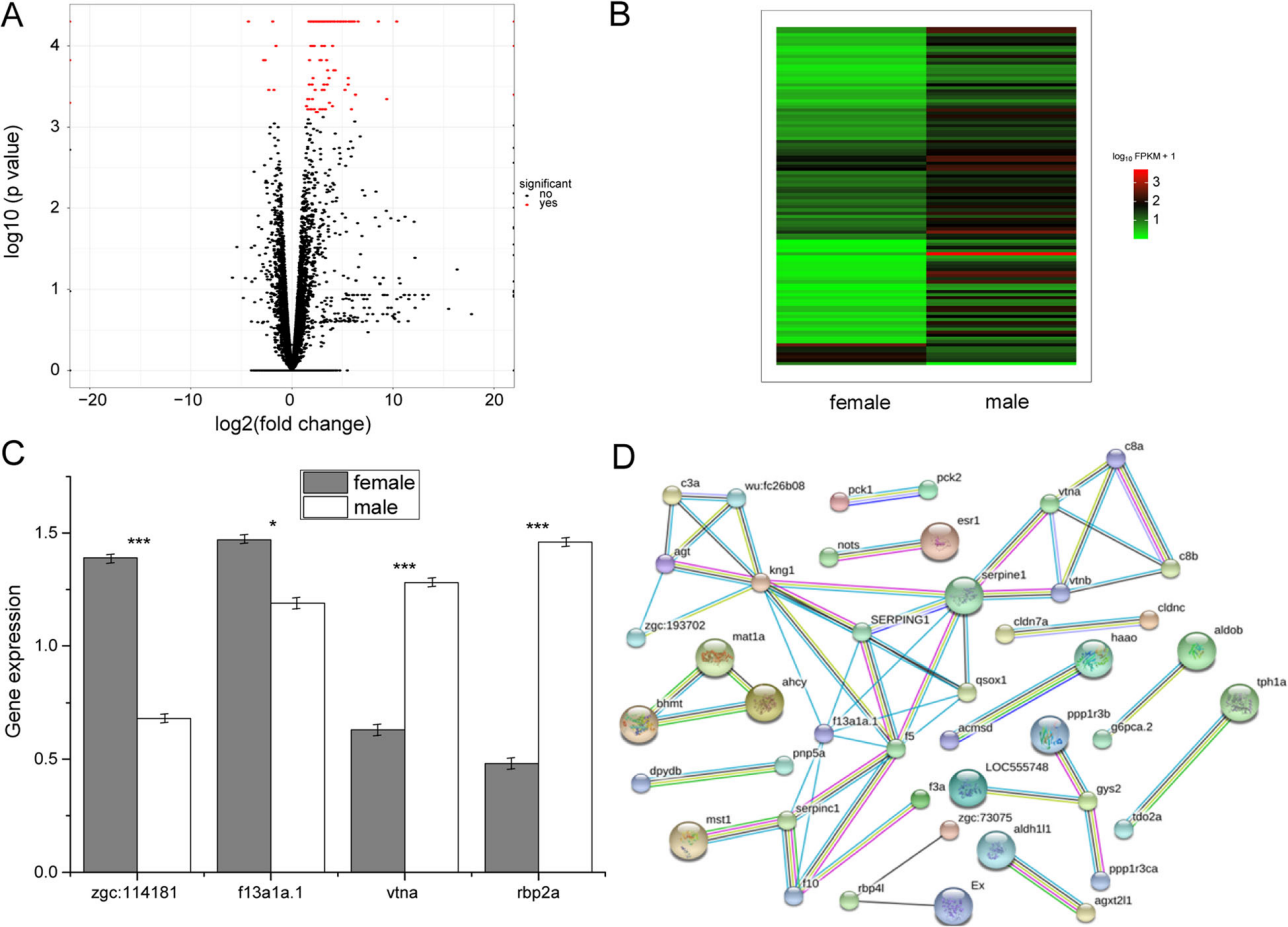
Characterization and verification of differentially expressed genes. a Volcano plots obtained by using fold-change values and *P*-values; (b) Hierarchical clustering analysis of mRNAs that are differentially expressed between the female and male zebrafish samples. Each group contains three individuals and the expression values are represented in different colors; (c) Significantly differentially expressed genes by RT-qPCR. Gray and white bars represent the female and males, respectively. The error bars represent standard error (***, *P* < 0.001; and *, *P* = 0.05); and (d) Protein-protein interaction network. The green edges represent gene neighborhood, the red edges represent gene fusions, the blue edges represent gene co-occurrence, the black edges represent gene co-expression, and the yellow edges represent text mining

We analyzed the transcriptome data of the six zebrafish brain tissue samples to obtain the sex-based gene expression of zebrafish in brain. Using the TopHat2 and Cufflinks packages, 14,315 annotated genes were obtained, accounting for 93.4% of the total genes assembled in the danRer10 zebrafish genome. Volcano and hierarchical clustering showed that the sex-based gene expression levels were distinguishable and statistically significant (Fig. 1a, b). We identified seven female-based genes and 101 male-based genes (fold-change > 2 and *P*-value < 0.05) (Additional file 2). When taking into account the direction of expression, approximately 93% (101/108) of the differentially expressed genes showed male-based expression (Additional file 2). Through literature search, we found that the detailed interpretation for majority of the differentially expressed male-based genes were not available. The expression levels of f13a1a.1, zgc:114181 and hbaa2 were up-regulated in the female zebrafish by 4.3, 2.8 and 2.6-folds, respectively, while apoa2, leg1.1, and c3a.1 were down-regulated by 10.37, 9.38 and 8.54-folds, respectively. We randomly selected four differentially expressed genes (zgc: 114181, f13a1a.1, vtna and rbp2a, 2 up-regulated and 2 down-regulated) for RT-qPCR verification, with the results indicating that the expression pattern showed a similar trend between RNA-seq and RT-qPCR results (Fig. 1c).

To verify the interrelationship between the differentially expressed genes, we constructed a protein-protein interaction network of these regulated genes with 44 nodes and 54 interactions (Fig. 1d). In this network, kininogen 1 (kng1, degree = 8, the degree of a node is the number of edges connecting to other nodes), serpin peptidase inhibitor clade E 1 (serpine1 degree = 7) and coagulation factor XIII A1 polypeptide a tandem duplicate1 (f13a1a.1, degree = 7) indicated higher degrees.

### Gene ontology and KEGG analyses

**Fig. 2:**
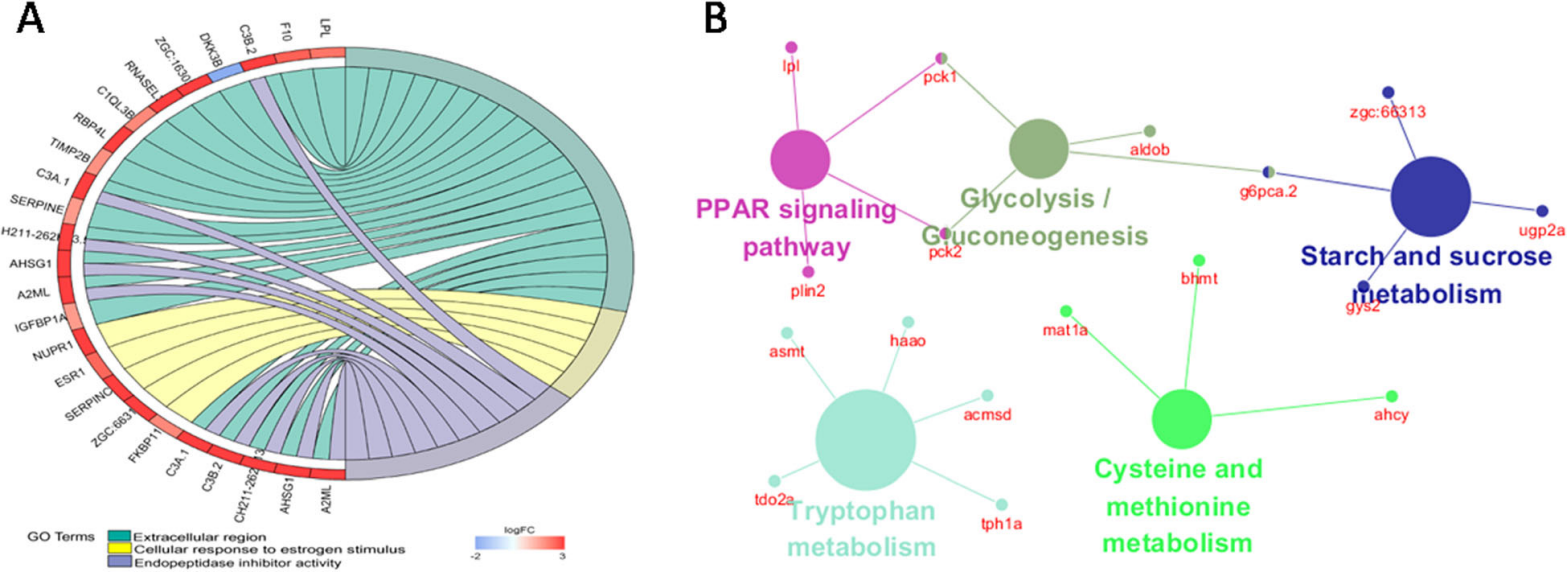
GO and KEGG analyses of differentially expressed genes. a GO enrichment analysis of the differentially expressed genes. Different colors represent the different GO classification entries; (b) Enriched pathway of the differentially expressed genes. The dots represent the genes, and the elliptical nodes represent the enriched pathway

To investigate the function of the differentially expressed sex-based genes, GO enrichment analysis and KEGG pathway annotation were performed. GO analysis showed that these differentially expressed sex-based genes were generally associated with the extracellular region, cellular response to estrogen stimulus and endopeptidase inhibitor activity (fold-change > 2 and FDR < 0.05, Fig. 2a). We found that the enrichment degree of the male-bias genes in gene ontology was significantly higher than that of the female-biased genes. These gene ontology terms may be associated with the observed behavioral differences between genders [19].

The KEGG analysis showed that the differentially expressed mRNAs remarkably enriched in PPAR signaling pathway, glycolysis/gluconeogenesis, starch and sucrose metabolism, tryptophan metabolism, and cysteine and methionine metabolism pathways (Fig. 2b). Previous studies found that PPAR signaling pathway plays important roles in mammalian reproductive system during the processes of the ovarian cycle, luteal formation, embryo implantation, placentation and male reproduction [20].

### Identification and characterization of long non-coding RNA

**Fig. 3:**
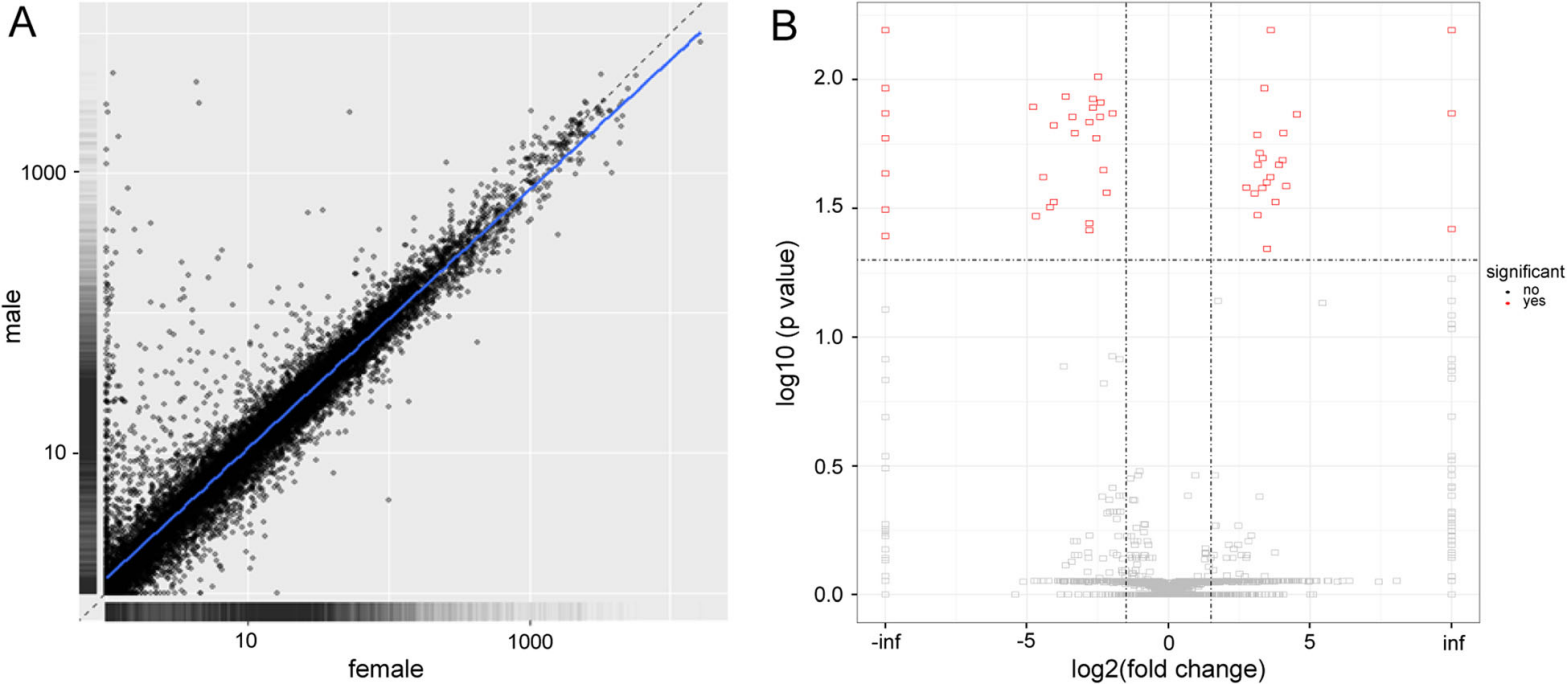
Characterization and verification of the differentially expressed lncRNAs. a the scatter plot of the lncRNA expression in the female and male zebrafish samples; (b) The volcano plots analysis of the lncRNAs that are differentially expressed between the female and male zebrafish samples. Inf indicates that the FPKM value of the gene in female zebrafish is 0. -inf indicates that the FPKM value of the gene in male zebrafish is 0

To investigate the biological function of the sex-related lncRNAs in zebrafish brain, the lncRNAs were identified in this study. Using coding prediction and ORF (open reading frame) identification software, we found that 3709 potential lncRNAs were expressed in the six zebrafish samples. Then the cuffdiff software was employed to analyze the potential lncRNAs, with a result of 28 female-based and 22 male-based differentially expressed lncRNAs (|log2(fold-change)| > 1.5, *P*-value < 0.05) (Additional file 3). The scatter plot showed a high degree of positive correlation (*P* = 0.913) between lncRNA expression in the female and male zebrafish samples (Fig. 3a). The volcano plots showed the differential expression levels of the lncRNA in the female and male samples (Fig. 3b).

### LncRNAs-mRNAs co-expression

**Fig. 4:**
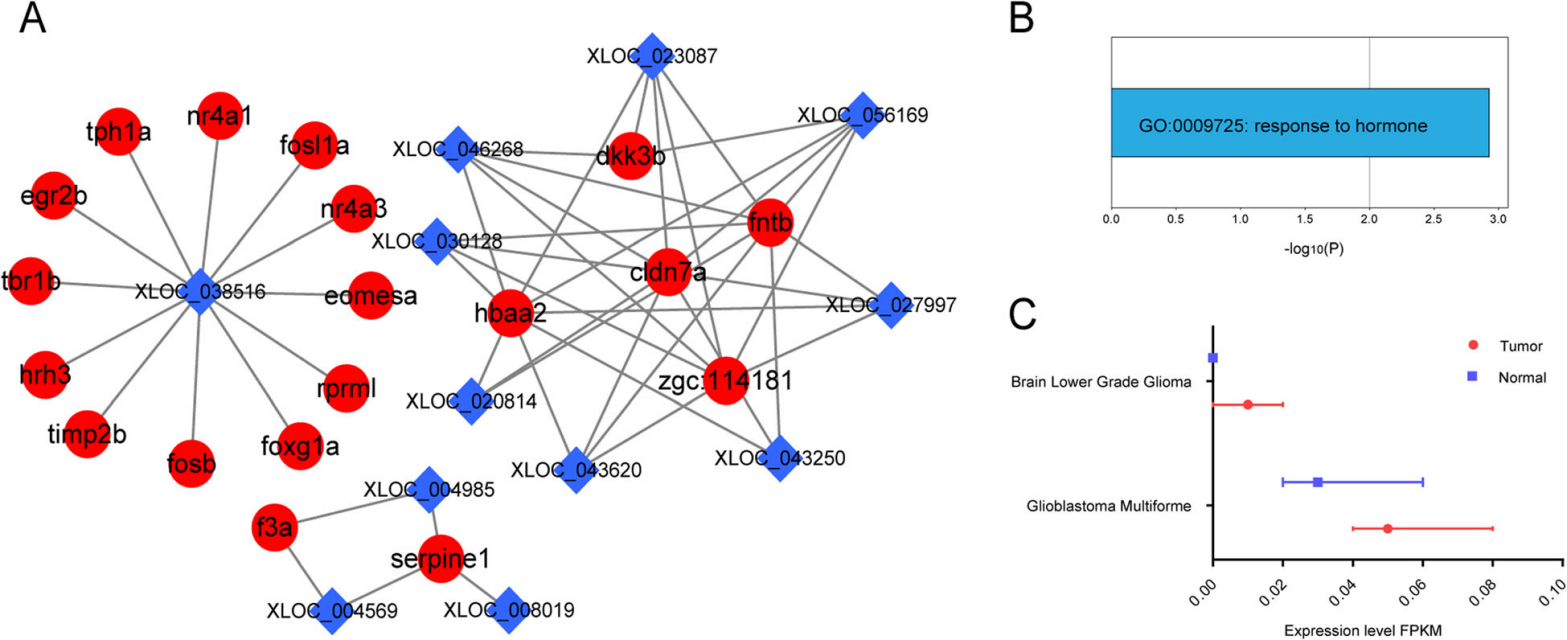
(**a**) LncRNAs-gene co-expression networks. A red circle represents a gene, whereas a blue diamond represents a lncRNA; (**b**) GO analysis of mRNA in co-expressed networks; (**c**) Expression of HSPA8P5 in several human diseases, data from TCGA database

To examine the collaboration between the lncRNAs and mRNAs in the gender-based zebrafish brain tissues, a co-expression analysis of the differentially expressed lncRNAs and the corresponding differentially expressed mRNAs was performed based on their FPKM values. The Pearson correlation coefficient between lncRNAs and mRNAs was calculated with the thresholds of absolute correlation coefficient |R| > 0.8 and *P* < 0.05, and significantly correlated lncRNA-mRNA couplings were obtained in 12 lncRNAs and 19 mRNAs (Fig. 4a). LncRNAXLOC_038516 (degree = 12) and XLOC_023087 (degree = 9) indicated higher degrees, and fntb (farnesyltransferase, CAAX box, beta) and hbaa2 (hemoglobin alpha adult 2) showed similar results. The lncRNA-mRNA coupling suggested that the regulation of cldn7a by multiple lncRNAs was likely to occur in the brain. Further GO analysis showed that mRNAs in the co-expression network were enriched on “response to hormone” (Fig. 4b).

The information of the 12 lncRNAs in the lncRNA–gene networks was shown in Table 1. For each of these lncRNAs, we found that the mRNA co-expressed with them was not within 300 kb from the same chromosome, indicating that they did not have *cis* regulatory functions and were not directly involved in the regulation of gene transcription or post-transcriptional levels. The ZFLNC database was used to perform a conservative analysis of the lncRNAs in the co-expression network. We found that XLOC_038516 and human pseudogene HSPA8P5 were considered as orthologs. Further analysis showed that HSPA8P5 was differentially expressed in multiple human brain neurological diseases (Fig. 4c).

**Table 1:** Information of 12 lncRNAs in lncRNA-gene networks

LncRNA	Chromosome	Regulation^a^	log_2_(FC)	*p*value
XLOC_004569	chr11:14098307–14,098,777	Up	2.76891	0.0421
XLOC_004985	chr11:38600939–38,601,149	Up	2.05469	0.0476
XLOC_008019	chr12:48960587–48,964,182	Up	5.43661	0.0012
XLOC_020814	chr19:17458973–17,459,464	Down	−3.31062	0.04015
XLOC_023087	chr2:31617390–31,623,697	Down	−2.7921	0.0258
XLOC_027997	chr21:32311853–32,312,662	Down	−1.72466	0.00235
XLOC_030128	chr22:24451902–24,454,348	Down	−1.98589	0.00215
XLOC_038516	chr3:25989342–25,989,841	Up	2.46855	0.02155
XLOC_043250	chr5:16082283–16,083,083	Down	−1.77434	0.0274
XLOC_043620	chr5:31375274–31,382,222	Down	−1.81491	0.01585
XLOC_046268	chr6:9650891–9,651,332	Down	−2.83877	0.0378
XLOC_056169	chrUn_KN150103v1:1249–2053	Down	−2.08877	0.01365

^a^Up- or down-regulated expression compared to expression levels in the female zebrafish

*FC* fold change

## Discussion

The zebrafish, as a model animal, has a nearly 70% similarities in genes between its genome and human genome [21], so exploring its brain-related dimorphism not only expands our understanding of the interaction between its reproductive processes and environmental stressors, but also has a positive effect on the analysis of human brain diseases [22].

In this study, we investigated the differential gene expression between 8-month-old male and female zebrafish brain tissues by using RNA-Seq sequencing technique, with a result of differentially expressed seven female-based genes and 101 male-based genes (fold-change > 2, and *P*-value < 0.05). These differentially expressed genes account for less than 1% of all genes identified from the brains of the male and female zebrafish. By RNA-seq data analysis, we found that only 61 genes showed significant gender effects in all four strains [7]. Sreenivasan et al. generated a gonad-derived zebrafish cDNA microarray and only observed 24 candidate genes showing a sexual dimorphic pattern [23]. These studies indicated that most gene expression levels are not significantly different between male and female zebrafish. Therefore, we speculated that they should also be similar in both male and female brains. Santos et al. found that 7478 expressed genes does not show a clear separation of the individual transcriptomes according to gender, suggesting that gender is not the main determinant of the variation between individual brain gene expression profiles [5]. Later, same conclusion was reached by Wong RY et al., suggesting that behavioral and physiological gender differences may be more easily facilitated by other factors such as the hormonal, ecological, or social environment [7].

Available evidence showed that many genes in the list of 108 sex- based genes we obtained in this study involves in neural circuit or brain development, e.g., egr2b (early growth response 2b, also known as KROX20) [24], mych (myelocytomatosis oncogene homolog) [25], and hrh3 (histamine receptor H3, [26]). In addition, we found that some genes play a similar role in the brain of zebrafish and humans. Zhang T et al. reported that apoa2 (apolipoprotein A-II) is abundant in the zebrafish brain and may perform a function during embryonic development [27], whereas in human, mutations in the gene may lead to adult glioma [28] and meningioma [29]. We have found that some genes have not been thoroughly studied in zebrafish, but have been identified to be important in human brain neural networks and diseases. For example, kng1 (kininogen 1) demonstrated tumor suppression and anti-angiogenic properties in glioblastoma [30]. Aldh1l1 (aldehyde dehydrogenase 1 family member L1) was also identified as a new astrocyte-specific marker in the brain [31]. In addition, dkk3b (dickkopf WNT signaling pathway inhibitor 3b), belonging to the family of secreted wnt-inhibitors with conserved cysteine-rich domains, displays a specific role during neuronal differentiation [32] and encodes a vital intracellular regulator of cell proliferation [33]. However, the function of most sex-based genes we found in this study is still unknown.

When performing GO enrichment analyses, an interesting result was obtained in the differentially expressed genes. The result showed that five genes (NUPR1, ESR1, SERPINC1, ZGC: 66313, and FKBP11) enriched in cellular response to estrogen stimulus, but these five genes were expressed in the brain of the male zebrafish. There is evidence that male behavior requires estrogen signaling, and adult gonads of any sex can support male behavior [34]. Furthermore, previous work had also reported that the sexually dimorphic gene expression in the zebrafish does not correspond to specific pathways, from which we can ascertain that commonalities in their regulatory mechanisms have the sex determining pathways in mammals [8].

For each lncRNA locus, the 300 kb upstream and downstream protein-coding genes were identified as *cis*-acting target genes; however these genes were not differentially expressed in our study. In the lncRNAs and mRNAs co-expression networks, some of the differentially expressed genes were previously reported to be involved in neural circuit such as egr2b and hrh3, indicating that lncRNA XLOC_038516 and XLOC_038516 may also have the same function. Further analysis revealed that HSPA8P5, one of the orthologs of XLOC_038516, was differentially expressed in multiple human brain neurological diseases. However, these conclusions have some limitations because all results are obtained in silicon. Further in vivo studies will help to fully understand the role of these lncRNAs in the brain of zebrafish.

## Conclusion

In this study, the mRNAs and lncRNAs with the sex-based differential expression were screened by transcriptome sequencing (RNA-seq) in the zebrafish brains. Based on the various biological analyses, we found that 12 new lncRNAs have significant gender specificity in the brain of zebrafish by analyzing the biological functions of the co-expressing mRNA. Our finding may provide a clue to further study of the functional difference between male and female zebrafish brain.

## Methods

### Acquisition of the Zebrafish Specimens

The wild-type AB zebrafish were purchased from the Wuhan Institute of Hydrobiology, Chinese Academy of Sciences, China, and the adult zebrafish of the same size (3 male and 3 female) were selected as experimental specimens.

### Animal euthanasia of zebrafish

We followed the NIH guidelines for zebrafish euthanasia (https://oacu.oir.nih.gov/sites/default/files/uploads/arac-guidelines/zebrafish.pdf). Immobilization by submersion in ice water (5 parts ice/1 part water, 0–4 °C) for 25 min following cessation of opercular movement. The fish were confirmed death by hypoxia after cessation of all movement. In this process, MS-222 solution (tricaine methane sulfonate, 168 mg/l) was used as anesthetic, which was buffered with sodium bicarbonate to pH = 7 before immersing the fish. The fish were left in the solution for 15 min following cessation of opercular movement. After anesthesia with MS222, the fish were frozen quickly in liquid nitrogen.

### Total RNA extraction and quality testing

Total RNA was extracted using Invitrogen Ambion RNA Extraction Kit according to the manufacturer’s protocol (ThermoFisher Scientific, MA, USA). RNA degradation and contamination were monitored on a 1% agarose gel. The RNA integrity number (RIN) was measured using an Agilent Bioanakyzer 2100 (Agilent Technologies, CA, USA) (Agilent, CA, USA) to assess the RNA quality. Sequencing was performed if the samples RIN values were greater than eight. The total RNA concentration was determined using a Qubit 2.0 fluorometer (Life Technologies, CA, USA).

### Sequencing library preparation, RNA-seq sequencing, and raw data preprocessing

Library Preparation was created using VAHTS Stranded mRNA-seq Library Prep Kit according to the manufacturer’s protocol (Vazyme, Nanjing, China). After the RNA samples passed the quality test, 2 μ g total RNA was enriched by magnetic beads with Oligo (dT). Subsequently, the Frag/Prime Buffer was used to break the mRNA into short fragments at 85 °C for 6 min. RNA fragments were converted to cDNA using random primers, followed by second-strand cDNA synthesis and end repair. The double-stranded cDNA was subsequently purified using AMPure XP beads (Beckman Coulter, CA, USA). The purified double-stranded cDNA was added an A and ligated to the sequencing linker, and AMPure XP beads were used for size selection of adapter-ligated DNA. Finally, PCR amplification was performed and the PCR product was purified using AMPure XP beads to obtain the final library. After the library was constructed, preliminary quantification was performed using Qubit 2.0 (Thermos fisher Scientific, MA, USA), and then the size of the library was detected using the DNA High Sensitivity DNA Kit (Bioanalyzer 2100, Agilent, CA, USA) to ensure the proper insert size of 350–450 bp. The concentration of the library was accurately quantified using KAPA Library Quantification Kits according to the manufacturer’s protocol (KAPA Biosystems, MA, USA). Subsequently, the library was sequenced using an Illumina HiSeq X Ten sequencing platform (Illumina, CA, USA). By using Trimmomatic software [35], Low-quality reads were filtered according to the following criteria: quality scores are less than 20, and reads with average quality scores of each read less than 20. FastQC software (http://www.bioinformatics.babraham.ac.uk/projects/fastqc/) [36] was employed to assess the quality of the raw reads.

### Mapping, annotation, and differential expression analysis for mRNA-seq data

The zebrafish reference genome (GRCz10/danRer10, Sep.2014) and the reference Index (the GTF file) were downloaded from UCSC (http://genome.ucsc.edu/). Firstly, bowtie2-build was used to index the reference genome, and then TopHat (version 2.1.0) was used [37] to map the reads to the reference genome. TopHat initially removed a small portion of the reads based on the quality information of each reads and then mapped the qualified reads to the reference genome [37]. In addition, the parameter of “--library– type” was set to “fr-firststr”, and the other parameters were set to default values. Then the result file of TopHat was input into Cufflinks software for further analyses, including transcript assembly, abundance estimation, and differential expression of RNA-Seq samples [38]. The confidence intervals for estimation of fragments per kilobase of transcript per million mapped reads FPKM were calculated using a Bayesian inference method [39]. Differential expressed genes were characterized according to the criterion of a fold change > 1.5 and q-value < 0.05.

### Gene ontology analyses and KEGG analysis

DAVID online tool (https://david.ncifcrf.gov) was used for identifying enriched biological themes [40]. Enriched GO terms with Gene-Count > 5 and *P*-value < 0.05 were selected as the thresholds for the subsequent analyses. Cytoscape software (two tools: ClueGO and CluePedia) was used for the Kyoto Encyclopedia of Genes and Genomes (KEGG) analysis [41], showing only pathways with P-value < 0.05. ClueGO network diagram was created based on Kappa statistics. Every node in the diagram represented a term that reflected the relationships between nodes, and the color of the nodes reflected the enrichment of the node classification.

### Validation by RT-qPCR

For the characterization of the zebrafish brain, RT-qPCR of mRNAs was performed using SYBR Green PCR Master Mix (Fermentas, Guangzhou, China) following the manufacturer’s instructions. The experiments were repeated at least in triplicate. The gene-specific primers were as follows:

zgc:114181(forward: 5′-TCACCGCCTTCCTCAGAAAT-3′;

reverse: 5′-ACTGAGCCGTGACCACTTTA-3′);

f13a1a.1(forward: 5′-GCGTGTCATTCCAAAACCCT-3′;

reverse: 5′-CAACTTGCACAGCCAGGATT-3′);.

vtna(forward: 5′-GACATTCGCCGGCTTGTATT-3′.

reverse: 5′-CAAGCGGACACTAAGGATGC-3′);.

rbp2a(forward: 5′-TGACTAAACAAAAGGGCGCC-3′;

reverse: 5′-CGCCTCTGTGCATCTTCTTC-3′).

β-actin(forward primer:5′-TCACCACCACAGCCGAAAG-3′;

reverse primer:5′-GGTCAGCAATGCCAGGGTA-3′).

β-actin was used as an internal control. The efficiencies of all sets of primers were between 91.6–97.3%. We used 96-hole RT-qPCR plates including three negative controls to discard false positive amplification signals. In each PCR plate, synthesized cDNAs without adding reverse transcriptase were used to confirm no genomic contamination. PCR reactions were performed at 95 °C for 5 min, followed by 40 cycles of 95 °C for 15 s and 60 °C for 1 min. The fold changes were calculated by ∆∆CT method [42].

### lncRNA identification and characterization

Cufflinks script was used to determine whether the detected transcripts were annotated by Refseq genes of zebrafish genome (build GRCz10/danRer10, Sep., 2014). Transcripts with length < 200 nt or > 10,000 nt were discarded, and only transcripts with exon number > 1 were retained. Coding Potential Calculator software (CPC, http://cpc.cbi.pku.edu.cn/) [43] and Coding Potential Assessment Tool (CPAT, http://lilab.research.bcm.edu/cpat/) [44] were used for the coding potential prediction analysis. Only transcripts that were considered “non-coding” by both of these tools were considered potential lncRNAs and software cuffdiff [38] was employed for subsequent analysis.

### Protein-protein interaction network and lncRNA-gene co-expression network construction

The STRING online software [45] was employed to construct the interaction network of the proteins encoded by the differentially expressed genes. A combined score > 0.7 was adopted as the cut-off criterion. According to the FPKM values of the different expression of the lncRNAs and the mRNAs in the six samples, the Pearson correlation coefficient between the lncRNAs and the mRNAs were calculated according to their FPKM values. The threshold for positive correlation was set to PCC > 0.8 and *P* < 0.05. Finally, the protein-protein interaction network and the regulatory network of lncRNAs-mRNA were established via Cytoscape software (http://www.cytoscape.org/) [41]. We used the ZFLNC database to conduct a conservative analysis of lncRNA [46].

### Statistical analysis

We used Pearson’s correlation coefficient to assess relationships between gene expression and stationary behavior and determined significance with two-tailed *P*-values, and a P-value of less than 0.05 was considered to be statistically significant.

In RT-qPCR validation experiment, a two-tailed t test was used to assess the statistical deference between male and female zebrafish, and a P-value of less than 0.05 was considered to be statistically significant.

Al the statistical analyses were performed using R (https://www.r-project.org/, version 3.5.1).

## Supplementary information

**Additional file 1.** The raw data files.

**Additional file 2.** Differentially expressed mRNAs in the brains of male and female zebrafish.

**Additional file 3.** Differentially expressed lnRNAs in the brains of male and female zebrafish.

## Data Availability

The data set generated and analyzed during this study is was submitted to Sequence Read Archive (SRA), with Project ID: PRJNA521037.

## Abbreviations

FPKM: Reads Per Kilobase of exon model per Million mapped reads
GO: Gene Ontology
KEGG: Kyoto Encyclopedia of Genes and Genomes
