# Supplementary material for: Transcriptome profiling analysis of sex-based differentially expressed mRNAs and lncRNAs in the brains of mature zebrafish (Danio rerio)
Source: BMC Genomics. 2019 Nov 8;20:830. doi: 10.1186/s12864-019-6197-9 (PMC6842217; doi:10.1186/s12864-019-6197-9)
Supplement: Supplementary file 2 — Additional file 2. Differentially expressed mRNAs in the brains of male and female zebrafish. [file 12864_2019_6197_MOESM2_ESM.docx]

| **Differentially expressed mRNAs in the brains of male and female zebrafish** | | | | | | | | | | | | | |
| --- | --- | --- | --- | --- | --- | --- | --- | --- | --- | --- | --- | --- | --- |
| test_id | gene_id | gene | locus | sample_1 | sample_2 | status | value_1 | value_2 | log2  (fold_change) | test_stat | p_value | q_value | significant |
| XLOC_009237 | XLOC_009237 | f13a1a.1 | chr24:2499152-2519576 | female | male | OK | 9.56845 | 0.485652 | -4.30029 | -3.80965 | 0.00005 | 0.004874 | yes |
| XLOC_004492 | XLOC_004492 | zgc:114181 | chr16:55296575-55320600 | female | male | OK | 424.798 | 60.8876 | -2.80256 | -2.56558 | 0.00015 | 0.012806 | yes |
| XLOC_010386 | XLOC_010386 | hbaa2 | chr3:54838982-54839797 | female | male | OK | 139.852 | 22.1954 | -2.65557 | -2.49976 | 0.00015 | 0.012806 | yes |
| XLOC_009923 | XLOC_009923 | dkk3b | chr25:16036174-16050445 | female | male | OK | 99.5912 | 20.383 | -2.28865 | -2.27481 | 0.00035 | 0.026745 | yes |
| XLOC_001080 | XLOC_001080 | nt5c2l1 | chr10:17502923-17527330 | female | male | OK | 48.763 | 13.0432 | -1.90249 | -2.70498 | 0.00005 | 0.004874 | yes |
| XLOC_013151 | XLOC_013151 | cldn7a | chr7:22345108-22355696 | female | male | OK | 43.8629 | 12.77 | -1.78024 | -2.0898 | 0.00035 | 0.026745 | yes |
| XLOC_007069 | XLOC_007069 | fntb | chr20:28901105-28924877 | female | male | OK | 91.8446 | 30.7078 | -1.58059 | -2.19641 | 0.0001 | 0.00897 | yes |
| XLOC_012640 | XLOC_012640 | mych | chr6:50452582-50455626 | female | male | OK | 25.633 | 69.3076 | 1.49501 | 2.04125 | 0.00055 | 0.039584 | yes |
| XLOC_002029 | XLOC_002029 | egr2b | chr12:8445994-8448481 | female | male | OK | 13.0392 | 38.539 | 1.56346 | 2.14335 | 0.00045 | 0.033158 | yes |
| XLOC_003410 | XLOC_003410 | plin2 | chr15:17094897-17112587 | female | male | OK | 15.3758 | 45.5779 | 1.56768 | 2.02577 | 0.0006 | 0.041035 | yes |
| XLOC_009160 | XLOC_009160 | nr4a1 | chr23:32224801-32231312 | female | male | OK | 78.2552 | 249.787 | 1.67444 | 2.12015 | 0.00045 | 0.033158 | yes |
| XLOC_005563 | XLOC_005563 | pdk2b | chr19:5756659-5769491 | female | male | OK | 25.7819 | 82.7152 | 1.68179 | 2.17424 | 0.00005 | 0.004874 | yes |
| XLOC_007299 | XLOC_007299 | igfbp1a | chr20:6818826-6822654 | female | male | OK | 57.6538 | 189.193 | 1.71437 | 2.14924 | 0.0003 | 0.023806 | yes |
| XLOC_013569 | XLOC_013569 | serpine1 | chr7:25994394-25998813 | female | male | OK | 12.1051 | 40.4929 | 1.74205 | 2.34224 | 0.00015 | 0.012806 | yes |
| XLOC_005856 | XLOC_005856 | eomesa | chr19:861017-867262 | female | male | OK | 9.0565 | 32.0043 | 1.82124 | 2.45704 | 0.00005 | 0.004874 | yes |
| XLOC_009881 | XLOC_009881 | tph1a | chr25:8015399-8035920 | female | male | OK | 8.14989 | 29.0079 | 1.83159 | 2.18453 | 0.0006 | 0.041035 | yes |
| XLOC_004013 | XLOC_004013 | nr4a3 | chr16:27410759-27433590 | female | male | OK | 26.5065 | 96.9724 | 1.87123 | 2.54512 | 0.0001 | 0.00897 | yes |
| XLOC_010788 | XLOC_010788 | timp2b | chr3:57562440-57595994 | female | male | OK | 4.95423 | 18.2243 | 1.87913 | 2.35234 | 0.00005 | 0.004874 | yes |
| XLOC_009350 | XLOC_009350 | f3a | chr24:31340189-31353497 | female | male | OK | 9.26278 | 35.3742 | 1.93318 | 2.24744 | 0.0006 | 0.041035 | yes |
| XLOC_007140 | XLOC_007140 | atf3 | chr20:37891798-37897858 | female | male | OK | 22.455 | 86.4412 | 1.94468 | 2.74286 | 0.00005 | 0.004874 | yes |
| XLOC_010586 | XLOC_010586 | hmox1a | chr3:25880612-25886721 | female | male | OK | 12.3192 | 48.9271 | 1.98972 | 2.26629 | 0.0003 | 0.023806 | yes |
| XLOC_012722 | XLOC_012722 | fkbp11 | chr6:7272044-7281508 | female | male | OK | 18.1341 | 74.0301 | 2.02941 | 1.99048 | 0.00045 | 0.033158 | yes |
| XLOC_006204 | XLOC_006204 | c1ql3b | chr2:3764153-3766794 | female | male | OK | 8.14188 | 33.3391 | 2.03378 | 2.33867 | 0.0001 | 0.00897 | yes |
| XLOC_005203 | XLOC_005203 | fosb | chr18:36788749-36793957 | female | male | OK | 57.9906 | 241.35 | 2.05724 | 2.63476 | 0.00005 | 0.004874 | yes |
| XLOC_013307 | XLOC_013307 | hrh3 | chr7:41468825-41471952 | female | male | OK | 8.68728 | 36.8981 | 2.08657 | 2.37491 | 0.00025 | 0.020495 | yes |
| XLOC_000249 | XLOC_000249 | rtn4rl2a | chr1:43789773-43792245 | female | male | OK | 64.6331 | 277.413 | 2.10169 | 2.61416 | 0.00005 | 0.004874 | yes |
| XLOC_014168 | XLOC_014168 | slc25a25a | chr8:2547523-2557585 | female | male | OK | 24.5393 | 107.268 | 2.12806 | 2.80249 | 0.00005 | 0.004874 | yes |
| XLOC_008379 | XLOC_008379 | rrbp1b | chr22:25059296-25082243 | female | male | OK | 3.47614 | 15.8446 | 2.18843 | 2.29901 | 0.00005 | 0.004874 | yes |
| XLOC_004453 | XLOC_004453 | si:dkey-33i11.3 | chr16:45282338-45321883 | female | male | OK | 1.48376 | 6.81574 | 2.19962 | 2.21221 | 0.0001 | 0.00897 | yes |
| XLOC_008135 | XLOC_008135 | vtnb | chr21:39022451-39035957 | female | male | OK | 15.0299 | 69.4749 | 2.20865 | 2.20268 | 0.0006 | 0.041035 | yes |
| XLOC_006740 | XLOC_006740 | si:dkeyp-73d8.6 | chr2:32638411-32641341 | female | male | OK | 11.598 | 56.838 | 2.29298 | 2.45201 | 0.00035 | 0.026745 | yes |
| XLOC_008312 | XLOC_008312 | lpl | chr22:15598250-15602858 | female | male | OK | 19.5725 | 98.0011 | 2.32397 | 3.13707 | 0.00005 | 0.004874 | yes |
| XLOC_006646 | XLOC_006646 | dpydb | chr2:20167149-20462201 | female | male | OK | 1.13891 | 6.07277 | 2.41469 | 2.32039 | 0.00065 | 0.043969 | yes |
| XLOC_006540 | XLOC_006540 | c8b | chr2:2387407-2401583 | female | male | OK | 4.00642 | 21.5183 | 2.42518 | 2.63778 | 0.00005 | 0.004874 | yes |
| XLOC_015032 | XLOC_015032 | tbr1b | chr9:51970052-51976287 | female | male | OK | 1.44303 | 7.76617 | 2.4281 | 2.58797 | 0.00005 | 0.004874 | yes |
| XLOC_012491 | XLOC_012491 | aldh1l1 | chr6:20008877-20041590 | female | male | OK | 7.66808 | 42.4693 | 2.46948 | 2.80551 | 0.00005 | 0.004874 | yes |
| XLOC_007401 | XLOC_007401 | esr1 | chr20:26460669-26484278 | female | male | OK | 4.19855 | 23.9101 | 2.50965 | 2.7547 | 0.00005 | 0.004874 | yes |
| XLOC_002288 | XLOC_002288 | haao | chr13:8626414-8650462 | female | male | OK | 3.39352 | 19.3377 | 2.51056 | 2.11862 | 0.00065 | 0.043969 | yes |
| XLOC_010153 | XLOC_010153 | rprml | chr3:19459897-19461239 | female | male | OK | 5.87272 | 33.5541 | 2.51439 | 2.80948 | 0.00005 | 0.004874 | yes |
| XLOC_000007 | XLOC_000007 | f10 | chr1:136019-142173 | female | male | OK | 5.04811 | 30.5901 | 2.59925 | 2.7713 | 0.00005 | 0.004874 | yes |
| XLOC_010842 | XLOC_010842 | slc38a4 | chr4:1680969-1717259 | female | male | OK | 9.51234 | 58.0613 | 2.6097 | 2.68753 | 0.00005 | 0.004874 | yes |
| XLOC_007896 | XLOC_007896 | bhmt | chr21:94307-99400 | female | male | OK | 18.3246 | 112.862 | 2.62271 | 2.73948 | 0.00005 | 0.004874 | yes |
| XLOC_003472 | XLOC_003472 | vtna | chr15:28269293-28276051 | female | male | OK | 2.59321 | 16.7735 | 2.69337 | 2.46106 | 0.00005 | 0.004874 | yes |
| XLOC_003094 | XLOC_003094 | pmt | chr14:1422192-1432756 | female | male | OK | 9.42768 | 60.9875 | 2.69354 | 2.57681 | 0.00005 | 0.004874 | yes |
| XLOC_008062 | XLOC_008062 | eif4ebp3 | chr21:26364636-26369550 | female | male | OK | 23.0927 | 155.078 | 2.74748 | 2.51682 | 0.00005 | 0.004874 | yes |
| XLOC_004592 | XLOC_004592 | ppp1r3ca | chr17:23291361-23293858 | female | male | OK | 1.88001 | 12.8268 | 2.77035 | 2.38247 | 0.00015 | 0.012806 | yes |
| XLOC_012151 | XLOC_012151 | il13ra2 | chr5:36501911-36516093 | female | male | OK | 1.3674 | 9.33339 | 2.77096 | 2.31114 | 0.0006 | 0.041035 | yes |
| XLOC_003395 | XLOC_003395 | diabloa | chr15:14918872-14922167 | female | male | OK | 10.6442 | 77.956 | 2.87259 | 2.77301 | 0.00005 | 0.004874 | yes |
| XLOC_014489 | XLOC_014489 | exorh | chr8:54189896-54191541 | female | male | OK | 3.84043 | 28.4279 | 2.88797 | 2.466 | 0.00015 | 0.012806 | yes |
| XLOC_013056 | XLOC_013056 | pnp5a | chr7:6517268-6532726 | female | male | OK | 6.90296 | 53.6439 | 2.95813 | 3.44196 | 0.00005 | 0.004874 | yes |
| XLOC_013600 | XLOC_013600 | nmbb | chr7:29814123-29815892 | female | male | OK | 6.10145 | 47.4966 | 2.9606 | 2.10201 | 0.0001 | 0.00897 | yes |
| XLOC_013121 | XLOC_013121 | si:ch73-71d17.1 | chr7:19137362-19149532 | female | male | OK | 0.916979 | 7.29608 | 2.99216 | 2.3063 | 0.00035 | 0.026745 | yes |
| XLOC_013379 | XLOC_013379 | etnppl | chr7:59774001-59785167 | female | male | OK | 0.849604 | 6.82692 | 3.00637 | 2.24982 | 0.0006 | 0.041035 | yes |
| XLOC_009512 | XLOC_009512 | slc51a | chr24:24816725-24837773 | female | male | OK | 1.64265 | 13.377 | 3.02566 | 2.56769 | 0.00005 | 0.004874 | yes |
| XLOC_010113 | XLOC_010113 | nupr1 | chr3:15355472-15356357 | female | male | OK | 13.9955 | 118.988 | 3.08778 | 3.01033 | 0.00005 | 0.004874 | yes |
| XLOC_003314 | XLOC_003314 | zmp:0000000758 | chr14:47170062-47185973 | female | male | OK | 1.23632 | 11.0741 | 3.16307 | 2.57061 | 0.0003 | 0.023806 | yes |
| XLOC_000296 | XLOC_000296 | ugp2a | chr1:50353356-50371828 | female | male | OK | 0.910319 | 8.23786 | 3.17783 | 2.51641 | 0.00035 | 0.026745 | yes |
| XLOC_004697 | XLOC_004697 | zgc:66313 | chr17:43754675-43773231 | female | male | OK | 4.10834 | 37.2123 | 3.17915 | 3.1398 | 0.00005 | 0.004874 | yes |
| XLOC_014689 | XLOC_014689 | tfcp2l1 | chr9:38483151-38501952 | female | male | OK | 1.52963 | 14.1873 | 3.21334 | 2.81221 | 0.0001 | 0.00897 | yes |
| XLOC_003225 | XLOC_003225 | fosl1a | chr14:30401239-30407372 | female | male | OK | 15.7448 | 150.657 | 3.25832 | 3.87662 | 0.00005 | 0.004874 | yes |
| XLOC_015050 | XLOC_015050 | asmt | chr9:56380374-56395051 | female | male | OK | 0.757685 | 7.30455 | 3.26913 | 2.18368 | 0.0006 | 0.041035 | yes |
| XLOC_007994 | XLOC_007994 | ppp1r3b | chr21:19879823-19883231 | female | male | OK | 0.448347 | 4.46125 | 3.31476 | 2.22636 | 0.0006 | 0.041035 | yes |
| XLOC_008711 | XLOC_008711 | slc34a2b | chr23:1068951-1094122 | female | male | OK | 0.747891 | 8.11204 | 3.43916 | 2.70841 | 0.00015 | 0.012806 | yes |
| XLOC_008638 | XLOC_008638 | c3b.2 | chr22:26109347-26155340 | female | male | OK | 0.830282 | 9.03097 | 3.44321 | 2.98562 | 0.00005 | 0.004874 | yes |
| XLOC_008317 | XLOC_008317 | serpinc1 | chr22:15995940-16001921 | female | male | OK | 4.63128 | 50.5636 | 3.44862 | 3.42797 | 0.00005 | 0.004874 | yes |
| XLOC_007331 | XLOC_007331 | si:dkey-239i20.2 | chr20:15184948-15191357 | female | male | OK | 0.541364 | 6.05708 | 3.48395 | 2.73368 | 0.0003 | 0.023806 | yes |
| XLOC_008055 | XLOC_008055 | cldnc | chr21:25717670-25719514 | female | male | OK | 0.80135 | 9.24992 | 3.52894 | 2.75039 | 0.0002 | 0.016728 | yes |
| XLOC_010148 | XLOC_010148 | ldlra | chr3:19149568-19171664 | female | male | OK | 0.670888 | 7.85559 | 3.54958 | 2.79569 | 0.00005 | 0.004874 | yes |
| XLOC_002624 | XLOC_002624 | mat1a | chr13:18497914-18506159 | female | male | OK | 1.3872 | 16.6881 | 3.58857 | 3.10137 | 0.00005 | 0.004874 | yes |
| XLOC_000004 | XLOC_000004 | zgc:163025 | chr1:109797-119138 | female | male | OK | 0.340536 | 4.32759 | 3.66769 | 2.79561 | 0.00025 | 0.020495 | yes |
| XLOC_014894 | XLOC_014894 | acmsd | chr9:23433424-23443001 | female | male | OK | 0.58216 | 7.61556 | 3.70946 | 2.52229 | 0.0005 | 0.036409 | yes |
| XLOC_012992 | XLOC_012992 | mst1 | chr6:53393745-53426394 | female | male | OK | 1.56892 | 20.9898 | 3.74185 | 3.08961 | 0.00005 | 0.004874 | yes |
| XLOC_004901 | XLOC_004901 | foxg1a | chr17:29100063-29102374 | female | male | OK | 0.868477 | 11.8639 | 3.77195 | 3.52301 | 0.00005 | 0.004874 | yes |
| XLOC_014664 | XLOC_014664 | f5 | chr9:34317819-34338811 | female | male | OK | 1.18041 | 16.8793 | 3.83789 | 3.54783 | 0.00005 | 0.004874 | yes |
| XLOC_007388 | XLOC_007388 | cyp2ad2 | chr20:25635581-25643081 | female | male | OK | 3.02887 | 43.6126 | 3.84789 | 3.57892 | 0.00005 | 0.004874 | yes |
| XLOC_012636 | XLOC_012636 | ahcy | chr6:49927461-49967635 | female | male | OK | 33.4978 | 537.84 | 4.00504 | 2.90798 | 0.00055 | 0.039584 | yes |
| XLOC_005611 | XLOC_005611 | rcvrn3 | chr19:10420612-10433651 | female | male | OK | 0.476038 | 7.7218 | 4.01979 | 3.24282 | 0.0001 | 0.00897 | yes |
| XLOC_002927 | XLOC_002927 | rtn4rl2b | chr14:16891478-16894137 | female | male | OK | 6.85566 | 113.915 | 4.05452 | 4.43367 | 0.00005 | 0.004874 | yes |
| XLOC_011294 | XLOC_011294 | gys2 | chr4:16811045-16835337 | female | male | OK | 0.332312 | 5.85136 | 4.13816 | 3.03969 | 0.0002 | 0.016728 | yes |
| XLOC_005371 | XLOC_005371 | ces2 | chr18:17419413-17426531 | female | male | OK | 2.91409 | 53.2468 | 4.19158 | 3.66947 | 0.00005 | 0.004874 | yes |
| XLOC_014357 | XLOC_014357 | gstt1a | chr8:30778380-30781887 | female | male | OK | 1.95783 | 35.8313 | 4.19389 | 3.44988 | 0.00005 | 0.004874 | yes |
| XLOC_009314 | XLOC_009314 | hgd | chr24:23814377-23825869 | female | male | OK | 2.38134 | 44.5075 | 4.22421 | 3.71725 | 0.00005 | 0.004874 | yes |
| XLOC_001522 | XLOC_001522 | gamt | chr11:5858691-5868283 | female | male | OK | 3.7212 | 71.9842 | 4.27384 | 3.2182 | 0.0002 | 0.016728 | yes |
| XLOC_008432 | XLOC_008432 | ahsg1 | chr22:37920961-37927975 | female | male | OK | 9.58004 | 214.715 | 4.48625 | 3.93919 | 0.00005 | 0.004874 | yes |
| XLOC_003719 | XLOC_003719 | a2ml | chr15:21178232-21196748 | female | male | OK | 1.61461 | 36.9627 | 4.51681 | 4.20028 | 0.00005 | 0.004874 | yes |
| XLOC_007998 | XLOC_007998 | rbp4l | chr21:20303053-20305386 | female | male | OK | 3.11175 | 82.8263 | 4.73429 | 4.46727 | 0.00005 | 0.004874 | yes |
| XLOC_014235 | XLOC_014235 | qsox1 | chr8:14465904-14517080 | female | male | OK | 1.26178 | 34.2059 | 4.76071 | 4.16532 | 0.00005 | 0.004874 | yes |
| XLOC_009450 | XLOC_009450 | pck2 | chr24:12775600-12794135 | female | male | OK | 0.485907 | 13.5716 | 4.80377 | 3.76787 | 0.00005 | 0.004874 | yes |
| XLOC_010052 | XLOC_010052 | zgc:172051 | chr3:1236409-1244024 | female | male | OK | 0.34267 | 9.96692 | 4.86226 | 3.49952 | 0.00005 | 0.004874 | yes |
| XLOC_010532 | XLOC_010532 | si:ch73-141c7.1 | chr3:17874239-17881055 | female | male | OK | 3.11638 | 104.742 | 5.07083 | 4.3611 | 0.00005 | 0.004874 | yes |
| XLOC_002373 | XLOC_002373 | agt | chr13:23858002-23865424 | female | male | OK | 2.00918 | 69.2451 | 5.10703 | 3.9093 | 0.00005 | 0.004874 | yes |
| XLOC_008699 | XLOC_008699 | si:ch211-262h13.5 | chr22:38825803-38832821 | female | male | OK | 0.785573 | 29.8726 | 5.24893 | 3.67257 | 0.00035 | 0.026745 | yes |
| XLOC_000693 | XLOC_000693 | sid4 | chr1:57971070-57980853 | female | male | OK | 1.39448 | 55.8233 | 5.32307 | 4.20397 | 0.00005 | 0.004874 | yes |
| XLOC_012680 | XLOC_012680 | pck1 | chr6:60060317-60067174 | female | male | OK | 1.79169 | 71.9311 | 5.32722 | 4.30878 | 0.00005 | 0.004874 | yes |
| XLOC_003647 | XLOC_003647 | rbp2a | chr15:5815658-5827065 | female | male | OK | 5.17048 | 244.897 | 5.56573 | 3.2996 | 0.0003 | 0.023806 | yes |
| XLOC_006186 | XLOC_006186 | c8a | chr2:2374699-2387109 | female | male | OK | 0.780702 | 37.1861 | 5.57385 | 3.4538 | 0.00025 | 0.020495 | yes |
| XLOC_008683 | XLOC_008683 | kng1 | chr22:36898337-36915609 | female | male | OK | 2.60597 | 128.44 | 5.62313 | 4.71995 | 0.00005 | 0.004874 | yes |
| XLOC_002933 | XLOC_002933 | aldob | chr14:20809209-20817949 | female | male | OK | 5.59992 | 279.953 | 5.64363 | 4.74921 | 0.00005 | 0.004874 | yes |
| XLOC_003022 | XLOC_003022 | tdo2a | chr14:36397759-36410270 | female | male | OK | 2.36849 | 122.911 | 5.6975 | 4.34375 | 0.00005 | 0.004874 | yes |
| XLOC_010640 | XLOC_010640 | rcn3 | chr3:32405205-32409308 | female | male | OK | 2.54164 | 145.081 | 5.83496 | 4.50567 | 0.00005 | 0.004874 | yes |
| XLOC_003024 | XLOC_003024 | rnasel3 | chr14:38313990-38315732 | female | male | OK | 0.912761 | 54.0697 | 5.88844 | 4.16591 | 0.0006 | 0.041035 | yes |
| XLOC_002603 | XLOC_002603 | adh8a | chr13:12521498-12527889 | female | male | OK | 2.86499 | 183.925 | 6.00444 | 4.79156 | 0.00005 | 0.004874 | yes |
| XLOC_015080 | XLOC_015080 | nots | chrUn_KN149830v1:6655-16932 | female | male | OK | 1.54196 | 110.924 | 6.16866 | 4.50682 | 0.00005 | 0.004874 | yes |
| XLOC_014220 | XLOC_014220 | serping1 | chr8:13922949-13934921 | female | male | OK | 0.745612 | 58.3689 | 6.29063 | 3.79229 | 0.0004 | 0.030193 | yes |
| XLOC_001752 | XLOC_001752 | g6pca.2 | chr12:6007977-6014702 | female | male | OK | 2.41863 | 226.805 | 6.55112 | 3.95263 | 0.00005 | 0.004874 | yes |
| XLOC_000682 | XLOC_000682 | c3a.1 | chr1:55460925-55507674 | female | male | OK | 0.720796 | 269.593 | 8.54697 | 4.98064 | 0.00005 | 0.004874 | yes |
| XLOC_006918 | XLOC_006918 | leg1.1 | chr20:1391531-1400179 | female | male | OK | 0.587867 | 392.392 | 9.38259 | 6.10493 | 0.00045 | 0.033158 | yes |
| XLOC_003984 | XLOC_003984 | apoa2 | chr16:24063945-24065236 | female | male | OK | 3.37741 | 4472.21 | 10.3709 | 6.20427 | 0.00005 | 0.004874 | yes |
|  |  |  |  |  |  |  |  |  |  |  |  |  |  |
